# From control to elimination: the research agenda for measles

**Published:** 2010-05-12

**Authors:** Robin Biellik

**Affiliations:** 1 Consultant Epidemiologist, Geneva, Switzerland

**Keywords:** Measles, Elimination, Research, Control, Vaccine

## Editorial

Research on measles virology, epidemiology, vaccine and vaccination strategies has expanded in recent years. Measles mortality reduction has already contributed significantly to progress towards the achievement of the 4^th^ Millennium Development Goal to reduce under 5 mortality by two thirds by 2015 compared with 1990 levels [1]. Interest in measles has increased with the increasing number of WHO regions (currently 5 out of 6, all except Southeast Asia) which have set time-limited goals to move from regional measles mortality reduction to the regional elimination of indigenous transmission. Indeed, the WHO Executive Board has requested studies on the feasibility of global measles eradication as a first step towards the possible establishment of a global goal [2].

Despite the abundance of research, there are a number of unanswered questions as we move towards a goal of measles elimination, many of which will be critical to achieving the elimination goal.  We present here a list of research priorities from a programmatic and operational perspective.

### Measles epidemiology

In certain settings, susceptible adolescents and young adults have played a significant role in measles outbreaks [3]. Therefore, will the achievement of measles elimination require greater attention to immunizing susceptible adolescents and young adults? Are older susceptibles more or less significant in urban, rural or highly isolated settings?

WHO recommends that, where possible, HIV-positive infants and infants born to HIV-positive mothers should receive a measles dose at 6 months of age, in addition to the routine dose administered at 9 months of age [1]. Furthermore, in countries where an increasing number of HIV-positive children now receive anti-retroviral treatment and survive into adulthood, it is likely that secondary measles vaccine failure will begin to create significant new pools of susceptibles. In theory, the epidemiology of measles could alter in the absence of natural boosting by wild measles virus in highly vaccinated populations in the years following regional elimination and prior to global eradication (if that will eventually be achieved). If waning immunity is suspected in immunized populations, it will be necessary to verify immunity status through occasional sero-surveys. If waning immunity is indeed detected, will adolescent or adult booster doses of measles vaccines be indicated?

As measles transmission declines generally, the relative importance of nosocomial measles transmission will increase, and several nosocomial outbreaks have been identified in the past decade [4,5]. WHO guidelines for the prevention and control of nosocomial measles were established some years ago [6], but are not systematically implemented in many countries. Therefore, should greater emphasis be placed on vaccinating all infants attending out-patient clinics or admitted to hospital in communities where measles is circulating or where measles is considered likely to spread from neighbouring communities?

### Measles surveillance

Experience from other vaccine-preventable disease initiatives has shown that the establishment of case-based, laboratory-based surveillance is essential as we move from disease control to elimination. Therefore, what are the critical surveillance indicators for measuring progress toward elimination and maintenance of elimination?  What is the sensitivity and specificity of the proposed set of the “elimination certification” criteria and of each criterion alone? Is it epidemiologically useful and cost-effective to continue conducting extensive genetic characterization of vaccine viruses? Are there benefits to be reaped from showing a declining number of genetic lineages?

WHO currently recommends the use of measles IgM serology for case confirmation in surveillance in countries that adopt measles mortality reduction or elimination goals [8]. However, in recent years, alternative methods measuring measles IgG in dried blood-spots and oral fluid with improved sensitivity and specificity have become available that have demonstrated value for establishing population immunity to measles have become available [9]. These approaches are more acceptable because they avoid the need for venipuncture. The costs of specimen collection and laboratory assays for serum specimens, dried blood-spots and oral fluid are currently roughly similar [10]. Would it be operationally simpler to switch to oral fluid specimens for laboratory confirmation of measles?

### Vaccination strategies

We know that in developing countries where the first dose of measles vaccine is administered to infants <12 months of age, a second dose is required to eliminate susceptibles from the population and interrupt measles transmission [11]. Under field conditions, what levels of 1^st^ and 2^nd^ dose vaccination coverage will interrupt measles transmission?

In most developing countries, the 2nd measles dose is currently delivered through a mass campaign strategy that is repeated when a critical mass of new susceptibles is estimated to have entered the population [11]. Current practice is to space measles campaigns at intervals of three years. Can the current method for estimating the interval [12] be improved upon?

Furthermore, what is the ideal age, from both the epidemiological and operational perspectives, for the administration of the 2^nd^ dose? In the Mideast, the second routine dose is more often given on school entry than in the second year of life. Is this policy more widely applicable?

Increasingly, developing countries are considering delivering the 2^nd^ measles dose through the routine immunization programme, for operational convenience.  Are the same levels of 1^st^ and 2^nd^ dose vaccination coverage required to interrupt measles transmission, regardless of the delivery strategy adopted for the 2^nd^ dose?

Does measles vaccination coverage increase or decrease when mass measles campaigns are integrated with the delivery of other public health interventions such as insecticide Treated nets (ITN) distribution or with other contacts with eligible children such as Child Health Days? In particular, what are the possible advantages of giving measles vaccination at the same time as malaria bednet distribution?

Can very large countries, such as Nigeria, Indonesia, China and India, follow the same strategy as other countries, in particular simultaneous nationwide campaigns? Vaccinating 50 to 100 million children in a single week presents special challenges, both from the operational and from the vaccine supply standpoints.

The PAHO model for integrated measles and rubella elimination is applicable only where countries adopt the PAHO objective of regional rubella elimination. When countries introduce rubella vaccination, either for control or, as in the Americas, for elimination, what is the best approach to harmonizing approaches to rubella and measles vaccination?

The house-to-house approach is fundamental to polio eradication, but not, at present, to measles. Can countries with weak health infrastructures interrupt measles transmission without such practices as preregistration of vaccinees and house-to-house mobilization?

### Alternative vaccine delivery technologies

What are the possible policy changes if needleless measles vaccine, given as an aerosol, is licensed and commercialized? Will measles campaign approaches more closely resemble the door-to-door approach of polio campaigns? In the event that India, for example, licensed needleless technology, what field studies would be needed to fit the new technology into the existing strategies?

### Co-administration of measles vaccine with other vaccines

How does the co-administration measles vaccine with other vaccines (e.g., yellow fever, Japanese encephalitis, tetanus toxoid, etc...) affect vaccine safety and immunogenicity?

## Acknowledgements

The author gratefully acknowledges technical advice received from Robert Davis MPH, Measles Delegate, American Red Cross, Nairobi, Kenya.

## Competing interests

The author reports no conflicts of interest.

## References

[1] WHO (2008). Progress in global measles control and mortality reduction, 2000–2007. Wkly Epidemiol Rec..

[2] WHO. 2009 (2009). Executive Board 125th Session, agenda item 5.1. WHO/EB/125/4,.

[3] Pan-American Health Organization Update: Sao Paulo Measles Outbreak in EPI Newsletter. http://www.paho.org/nenglish/hvp/hvi/sne2001.pdf.

[4] Chen J, Tsou T, Liu D (2009). Measles resurgence in Taiwan – lessons learned. J Formos Med Assoc.

[5] Follin P, Dotevall L, Jertborn M, Khalid Y, Liljeqvist JA, Muntz S, Qvarfordt I, Söderström A, Wiman A, Ahrén C, Osterberg P, Johansen K (2008). Effective control measures limited measles outbreak after extensive nosocomial exposures in January-February 2008 in Gothenburg, Sweden. Euro Surveill.

[6] WHO. 1995 Measles control in the 1990s: minimising nosocomial transmission.. WHO publication WHO/EPI/GEN/95.4.

[7] Scott S, Mossong J, Moss WJ, Cutts FT, Cousens S (2008). Predicted impact of the HIV-1 epidemic on measles in developing countries: results from a dynamic age-structured model.. Int J Epidemiol..

[8] Guris G (2001). Module on best practices for measles surveillance.. WHO.

[9] Biellik RJ, Brown DW (2009). Measles mortality reduction in Africa.. Lancet..

[10] Measles and rubella laboratory network (2008). 2007 meeting on use of alternative sampling techniques for surveillance.. Wkly Epidemiol Rec..

[11] WHO-UNICEF (2001). Measles mortality reduction and regional elimination strategic plan 2001-2005..

[12] de Quadros CA, Olivé JM, Hersh BS, Strassburg MA, Henderson DA, Brandling-Bennett D, Alleyne GA (1996). Measles elimination in the Americas: Evolving strategies.. JAMA..

